# Paragangliomas of the Head and Neck: A Review of the Latest Diagnostic and Treatment Methods

**DOI:** 10.3390/medicina60060914

**Published:** 2024-05-30

**Authors:** Dragos Octavian Palade, Razvan Hainarosie, Adina Zamfir, Daniela Vrinceanu, Mihaela Pertea, Mihail Tusaliu, Florin Mocanu, Catalina Voiosu

**Affiliations:** 1 Surgery Department, Faculty of Medicine, “Grigore T. Popa” University of Medicine and Pharmacy, 700115 Iasi, Romania; 2 ENT Department, “Sf. Spiridon” Emergency Hospital Iasi, 700111 Iasi, Romania; 3 ENT Department, Faculty of Medicine, “Carol Davilla” University of Medicine and Pharmacy, 020021 Bucharest, Romania; 4 ENT Department, “Grigore Alexandrescu” Children’s Emergency Hospital, 011743 Bucharest, Romania

**Keywords:** head and neck, paraganglioma, neuroendocrine tumor

## Abstract

*Background and objectives*: Paragangliomas of the head and neck are rare, slow-growing neuroendocrine tumors, benign in their vast majority, but with a possibility of developing distant metastases. They show great inheritable character, and their behavior has proven to be unpredictable; therefore, they are considered malignant. *Material and methods:* This article aims to offer a more comprehensive presentation of the pathogenesis, epidemiology, diagnostic methods, imaging development, and treatment guidelines. We tried to bring together all the necessary data that, in our opinion, a head and neck practitioner should know when managing this type of tumor. Our main focus is on the most recent studies, with the purpose of a homogenous presentation of all current guidelines and approaches to this pathology. *Results*: Paragangliomas of the head and neck are still a disputed topic. One of the main reasons for that is their low incidence of 0.3 to 1 per 100,000 every year. The most frequent locations are the carotid body, the temporal bone, the jugular and mastoid foramen, and the vagal nerve. Their clinical presentation usually involves a painless lateral mass associated with symptoms such as hoarseness, hearing loss, tinnitus, and cranial nerve deficits. Up to 40% of them are inherited, mostly linked with mutations of succinate dehydrogenase complex. Imaging evaluation consists of CT and MRI, and new functional explorations such as ^18^F-FDA and ^18^F-FDG PET/CT, ^18^F-DOPA PET, ^123^I-MIBG, and ^68^Ga-DOTATE PET/CT. Measuring the catecholamine levels in the plasma and urine is mandatory, even though paragangliomas of the head and neck rarely display secretory behavior. Treatment mainly consists of surgery, with different approaches and techniques, but conservative management methods such as wait and scan, radiotherapy, proton therapy, and chemotherapy have proven their efficiency. The therapeutical decision lacks consensus, and current studies tend to recommend an individualized approach. Guidelines regarding long-term follow-up are still a matter of debate.

## 1. Introduction

Paragangliomas represent a rare group of tumors with a neuroendocrine origin that arises from the sympathetic and parasympathetic paraganglia [1]. The general consensus is that paragangliomas are, in their vast majority, benign tumors with a slow growth rate, 95% of them being non-secretory [2]. It has been uncovered that this set of tumors has an embryologic origin from the neural crest cells, being able to spread from the skull base even to the pelvis area [3]. Although paragangliomas display a heterogeneous genetic character, a set of genes seems to give them a hereditarian characteristic [4]. Paragangliomas represent the extra-adrenal counterpart to the pheochromocytoma [1].

Traditionally known as “glomus tumors” or “chemodectomas”, the World Health Organization categorized this designation to be obsolete, classifying them as non-epithelial neuroendocrine neoplasms (NEN) [5].

Head and neck paragangliomas represent a great chunk of all paragangliomas, with an estimated percentage between 65% and 70%, representing 0.65% of all tumors that occur at the head and neck level [1]. The incidence in the general population of the head and neck paragangliomas is estimated to vary between 0.3 to 1 per 100,000 [1]. These tumors usually tend to be discovered in female adults during their mid-life, in comparison with tumors with extra-adrenal origin located outside of the head and neck, which are more commonly found in male patients [6]. They constitute 3% of all head and neck tumors found every year, with a predominance of 3 to 4.1 of female patients aged between 40 and 70 years [7]. Head and neck paragangliomas are usually singular, unilaterally located, non-metastatic tumors [2].

The most common location for the head and neck paragangliomas is the carotid body, representing up to 60% [1]. They are more commonly situated at the bifurcation of the carotid artery within its adventitia [6]. They are followed by the temporal bone paragangliomas, constituting 20–30% of all paragangliomas, with a female predominance [8]. In third place comes the vagal nerve paragangliomas with 5% [9]. These tumors can also arise from more particular locations such as glossopharyngeal paraganglia, jugular-tympanic PGL, larynx, nasal cavity, trachea, thyroid gland, and orbit [10].

The vast majority of Paragangliomas, are considered to be benign tumors, but between 6% and 19% are proven to have a malignant behavior, being responsible for distant metastases [1]. Most metastases are usually found in the regional lymph nodes, but extensions to other sets of organs have also been discovered in 6% to 13% of the cases, the most common targets being the lungs and the skeleton [1]. It has also been proven that paragangliomas of the head and neck might be a source of hepatic metastases [11]. The lack of immunohistochemical criteria represents the primary challenge in differentiating benign from metastatic paragangliomas; only the identification of distant metastases can establish the diagnosis [7]. These are the reasons why the latest guidelines from the World Health Organization advise considering all paragangliomas to be malignant and be kept under strict observation even after surgical treatment is performed [5].

In recent years, paragangliomas have been recognized to have a genetic etiology and a high risk of heritability [10]. In 40% of the paraganglioma cases, a germline mutation of one of the following genes has been identified: VHL, SDHB, SDHD, RET, NF1, TMEM127, MAX, SDHC, SDHA, SDHAF2, HIF-2α, HRAS, KIF1B, PHD2, and FH [11]. Paragangliomas have been confirmed to be also linked with somatic mutations, such as RET, VHL, NF1, MAX, and HIF2A, that occur in 25% to 30%, later in life, and affect only a single cell from a particular tissue [11]. A close look at the genes in which mutations have been identified, and because 40% of all paragangliomas carry a genetic mutation, these tumors have been linked with multiple hereditary syndromes such as multiple endocrine neoplasia type 2, von Hippel-Lindau, neurofibromatosis type 1, and familial pheochromocytoma-paraganglioma syndrome [12]. Half of the cases with metastatic paragangliomas have been confirmed to be carriers of a mutation of the succinate dehydrogenase subunit B (SDHB) [13].

Histological and immunohistochemical analysis might offer important details for the diagnosis of paragangliomas and differentiate them from other types of tumors in the head and neck [5]. Paragangliomas of the head and neck usually present three histological aspects: (1) the Zellballen pattern, (2) the angiomatous pattern consisting of large spindle cells or crescent-shaped cells and capillaries, and (3) polyhedral cells with columnar architecture and abundant cytoplasm that form the adenomatous pattern [14]. According to the latest guidelines of the World Health Organization, the immunohistochemical examination of a specimen could offer important details such as a high Ki67, the presence of necrosis, which is associated with other aspects like a tumor larger than 5 cm, and the presence of SDHB mutation, which might indicate malignant behavior [5].

The clinical presentation of paragangliomas usually depends on the localization of the tumor [15]. Most commonly, they appear to be an asymptomatic neck mass, but in some cases, such as a vagal paraganglioma, which might associate palsies of the cranial nerves IX and XI, or when a middle ear paraganglioma is involved pulsatile tinnitus, hearing loss, and aural fullness could be present [15]. When it comes to a laryngeal paraganglioma, symptoms such as dyspnea, hoarseness, and stridor due to vocal fold paralysis might occur [16].

In evaluating the paragangliomas of the head and neck, imaging plays a crucial role. CT and MRI represent the first steps for an imagistic evaluation of the paragangliomas [17]. In cases of a jugular-tympanic or jugular glomus paraganglioma, a CT scan provides a better assessment of the contact of the tumor with the temporal bone, while an MRI provides a better description of the tumor involving the soft tissue [17]. Paragangliomas showed the highest uptake when assessing the SSTR expression, so the Ga-DOTATE PET/CT proved to be of use in differentiating them from other tumors such as schwannoma, meningioma, esthesioneuroblastoma, which proved to have a lower uptake [18].

The treatment conducted on the head and neck paragangliomas involves a full assessment of the family history and relatives’ DNA and genetic testing, localization and extent of the tumor, the existence of distant metastases, biological parameters, patient comorbidities, the experience of the surgical team, and a multidisciplinary evaluation [5]. Patients who cannot undertake either surgical or radiotherapy treatments and do not present a tumor with malignant behavior can benefit from a “watch and wait” strategy [5]. Nevertheless, surgery with large safety margins represents the primary treatment for head and neck paragangliomas in most circumstances, considering the benign nature of most of the tumors [19]. Patients who present a relapse of the tumors after a primary surgical intervention can benefit from the radiotherapy added to a second attempt to resect the tumor [19]. Patients who cannot undertake surgery due to associated comorbidities have the option of radiotherapy treatment [20].

## 2. Genetics

Recent studies on head and neck paragangliomas have shown that they have a genetic etiology, with up to 40% of them being associated with an identifiable genetic mutation [10]. This percentage boosts the paragangliomas to the first place in a ranking of genetic inherited tumors [10]. Genetic studies conducted on paragangliomas of the head and neck have identified 10 genes that are usually involved, such as four subunits of the succinate dehydrogenase complex (SDHA, SDHB, SDHC, and SDHD), succinate dehydrogenase assembly factor 2 (SDHAF2), the VHL gene involved in von Hippel–Lindau syndrome, neurofibromatosis type 1 (NF1), hypoxia-induced factor 2 alpha (HIF-2α), receptor tyrosine-protein kinase (RET) proto-oncogene, and transmembrane protein 12 (TMEM127) [11,21]. Other genes have also been confirmed to be playing an important role in the appearance of paragangliomas, such as EGLN1/PHD2 and EPAS/HIF2-α, which seem to point out that hypoxia might be a predisposal factor [12]. Therefore, the genetic mutations linked with paragangliomas have been spread into two main categories: Cluster 1, which includes tumors with a genetic mutation with involvement of the activation of the pseudohypoxia signaling pathway, such as VHL and SDHx; and Cluster 2 mutations, like RET/NF1/THEM127/MAX, that use the activation of the kinase pathway in their transcription process [12].

The World Health Organization’s latest guidelines advise the use of SDHB immunohistochemistry in all head and neck paragangliomas, as it is the most frequent mutation associated with cases of metastatic paragangliomas [5,10]. Although SDHB is the most frequent mutation confirmed in metastases associated with head and neck paragangliomas, SDHD is the most commonly encountered mutation of all four, mostly discovered in benign forms [22]. SDHD mutations presence seems to be associated with a higher risk of occult paragangliomas, and it is frequently linked with paraganglioma syndrome 1, which consists of a concomitant identification of pheochromocytoma [10,15,23]. At the same time, SDHC is usually confirmed in paraganglioma syndrome 3, and like SDHD, it is involved in less than 5% of cases with metastases [15]. The rarest of all paragangliomas-related syndromes are 5 and 2, being linked with SDHA and SDHAF2 [15]. It is worth mentioning that SDHA mutations are linked with gastrointestinal stromal tumor (GIST), being confirmed in almost 45% of all cases [10].

The international guidelines of screening for SDHx genetic mutations linked with paragangliomas strongly encourage the practice of periodically testing all patients and their relatives, even if they carry an asymptomatic paraganglioma or none at all [24].

## 3. Histopathology

The histological aspect of paragangliomas is represented by a chief cell organized in a nest-like pattern, called the “Zellballen” pattern, with a fibrovascular tissue that forms the stroma, and in the periphery, sustentacular cells [5,25]. Paraganglioma’s chief cells are most often epithelioid, and very rarely spindle, with round, hyperchromatic, and chromatin cluster nuclei [15]. These cells stain positive with synaptophysin, and their nuclei are amphophilic to pink [15]. The latest consensus from the World Health Organization considers biomarkers such as CD56, CD57, PGP9.5, and neuron-specific enolase to be obsolete and advises against using them in case of suspicion of paragangliomas [5]. The latest guidelines consider chromogranin-A to be the most specific biomarker, not only for paragangliomas but also for all neuroendocrine neoplasms [5,10]. Another biomarker that seemed to be practical for head and neck paragangliomas is GATA3, but due to the need for stronger antigen retrieval, it shows a large variation in expression detection, between 95% and 5% [26]. The structural support for the chief cells is provided by the sustentacular cells that surround them [13]. The histological specimen commonly lacks glandular cells and mucin but might display cellular pleomorphism and, in some cases, necrosis [13]. The mitotic activity is very rare, usually Ki67 being lower than 1 per 10 hpf [15]. There is no specific biomarker that can either confirm or deny the malignant behavior of the tumor [5].

When it comes to secretory head and neck paragangliomas, they tend to have a fluctuant reactivity to tyrosine hydroxylase and dopamine beta-hydroxylase, both enzymes being involved in the synthesis of catecholamines [27].

In need of a guideline, a Grading system for Pheochromocytoma and Paraganglioma (GAPP) has been proposed that takes into consideration the histological pattern, the presence of necrosis, any capsular or vascular invasion, cellularity, catecholamine type, and the Ki67 [28]. It offers a score that varies between 0 and 10, and classifies the tumors into well, moderate, and poorly differentiated, trying to establish a predictive algorithm for a malignant behavior with a sensitivity of 100% and a specificity of 68%, as shown in a recent study [29]. The World Health Organization neither endorses nor discourages the use of the GAPP score, since all paragangliomas have a potential malignant risk [5].

## 4. Clinical Presentation

The vast majority of head and neck paragangliomas are non-secretory tumors, therefore, the first challenge encountered by the physician when assessing the patient is the lack of specific symptoms [2]. Clinical characteristics depend mostly on the location of the tumor [1,10]. The typical presentation involves a painless neck mass that might cause hoarseness, tinnitus, neurological deficits related to the cranial nerve palsies, and pain in the ear [10,30].

The carotid body paragangliomas, which represent 60% of all cases of head and neck paragangliomas, are usually characterized by a pulsatile, painless neck mass located near the angle of the jaw, and the associated symptoms are due to compression it exhibits on the nearby cranial nerves like the vagus and glossopharyngeal nerve, being represented by hoarseness, dysphagia and autonomic dysfunctions [2,15].

The second most common location for a head and neck paraganglioma is the middle ear, with 30% of all, being usually characterized by pulsatile tinnitus, hearing loss, and aural fullness, appearing most commonly in the 6th life decade, with a metastatic risk of only 2% [15].

When it comes to vagal paragangliomas, they are usually associated with pulsatile tinnitus and neurological deficits such as shoulder weakness or Horner Syndrome, caused by encroaching other cranial nerves like IX, X, XI (spinal accessory branch), and XII [31].

## 5. Catecholamine Secreting

Head and neck paragangliomas have been traditionally considered to be non-secreting tumors in their vast majority, in contrast to those localized in the thorax or the abdomen, and the assumption for a secretory tumor has only been reserved for those with sympathetic origin [32]. It is estimated that between 3% to 4% of all paragangliomas secrete catecholamines, although all have the potential to produce it [33]. The suspicion that all head and neck paragangliomas might have the ability to produce catecholamines was raised when a study revealed them to be positive for tyrosine hydroxylase, which is an enzyme involved in the conversion of tyrosine in _L_-dihydroxyphenylalanine (_L_-DOPA), a precursor for dopamine [34]. The common symptoms that might indicate a secretory paraganglioma are tachycardia and hypertension, accompanied by the classical triad of headache, palpitations, and sweating [33].

Considering the fact that the main excretion pathway for catecholamines is through the kidney, the obvious target for determining any high levels is measuring the presence of metabolic products in the urine [33]. Commonly, metanephrine, normetanephrine, and vanillylmandelic acid are measured from the urine of the last 24 h [33]. Considering that catecholamines might be episodically secreted and that tumor cells were proven to constantly produce metanephrines via catechol-O-methyltransferase, recent studies pledge for using high-performance liquid phase chromatography for plasma-free metanephrine or normetanephrine, with a specificity of 96.1% and a sensitivity of 94.1% [35]. In cases of dopamine-secreting head and neck paragangliomas, studies showed that the best testing method is measuring the 3-methoxythyramine levels in plasma and urine [34,36].

Recent guidelines point out that failure to identify and treat high levels of catecholamine leads to significant morbidity and mortality; therefore, determining the secretory behavior of all head and neck paragangliomas has become mandatory before taking any other therapeutical action [32].

## 6. Imaging

Imaging represents a key component in the current management of head and neck paragangliomas, not only in establishing a diagnosis and describing the tumor anatomy and extension but also in monitoring postoperative patients and identifying possible distant metastases [10]. Imaging offers three major details about paragangliomas: primary tumor localization, the presence of multiple coexisting tumors allowing differentiation between an isolated mass and the possible implication of a syndromic paraganglioma, and the existence of distant metastases, being the sole method for their certain identification, allowing the medical team to decide between a curative treatment or a palliative one [17].

Obviously, the most accessible imaging method to assess any neck swelling is ultrasound, which highlights a solid mass with a heterogeneous hypoechoic structure; hypervascular under color flow Doppler, being useful for small paragangliomas, but extremely limited to give any other detail [37].

Paragangliomas on enhanced CT scan appear as a solid homogenous mass, with high uptake due to hypervascularization, the heterogeneity being present when necrosis and intra-tumoral hemorrhage or thrombosis are identified [25,37]. CT angiography offers key details regarding the anatomical relations with the internal carotid artery and venous return, allowing an extensive understanding of the vascularization of the tumor and offering guidance for surgical or chemoembolization decisions [25]. It has a sensitivity of 80–90% and a specificity of 90%, and highlights information about local bone invasion, in contrast to MRI, but is unreliable in the predicament of malignancy [17].

MRI has become the standard imaging method to assess paragangliomas, describing it as a solid mass, well delimited, with strong, heterogenous enhancement due to a rich vascular network, having a specific “salt-and-pepper” appearance, which translates into rapid flow vessel (the pepper) integrated in a tumoral matrix or bleeding zones with a high-intensity signal (the salt) [37]. MRI offers important details regarding the invasion of the surrounding soft tissue, having the same specificity and sensitivity as a CT scan, but involving no radiation, therefore being preferred to the latter [17].

In recent years, functional imaging methods have been developed, such as ^18^F-FDA and ^18^F-FDG PET/CT, ^18^F-DOPA PET, and ^123^I-MIBG, that specifically identify the paragangliomas using agents that target catecholamine synthesis, storage and secretion [38]. ^18^F-DOPA PET has a sensitivity of 90%, and a specificity of 79% for head and neck paragangliomas, being very sensitive—up to 100% for multifocal tumors [37]. The disadvantage of ^18^F-DOPA PET is the extremely low sensitivity for SDHB-related metastases— only 20%—while for non-SDHB ones, it performs extremely well, identifying up to 94% of them [38,39].

^123^I-MIBG scintigraphy shows a limited use for metastatic paragangliomas, identifying between 59 and 79% of them, but with a sensitivity of 92–98% for non-metastatic tumors [38].

^18^F-FDG PET/CT has great utility in localizing primary paragangliomas and metastases, especially those SDHB-related, but later results have shown fluctuating numbers for sensitivity, between 58 to 88%, so it should be interpreted with caution [17,38,39].

^18^F-FDA PET/CT has proven to be superior to ^18^F-DOPA PET and ^123^I-MIBG for identifying metastases and has offered great results, especially for pheochromocytomas and paragangliomas derived from the sympathetic nervous system, with a sensitivity and specificity between 70 and 95%, but unfortunately, it has performed poorly when it comes to paragangliomas of the head and neck, which are from the parasympathetic nervous system, revealing only 46% of them [17,39].

General recommendations encourage the use of ^18^F-DOPA PET and ^18^F-FDA PET/CT in patients with non-SDHB paragangliomas and restrict the ^18^F-FDG PET/CT in correlation with CT or MRI for patients with SDHB mutation [38,39].

^68^Ga-DOTATE PET/CT represents an incredibly valuable imaging resource in the evaluation of paragangliomas and their metastases due to the higher uptake related to the somatostatin receptor (SSTR) overexpression in this type of tumors, especially SSTR2, being able to detect 36% more lesions that CT or MRI [18]. Many studies show the superiority of ^68^Ga-DOTATE PET/CT over other functional imaging methods ^18^F-FDG PET/CT, ^18^F-FDA PET/CT, or ^18^F-DOPA PET being suggested that it should become the first choice in evaluating paragangliomas [18,40,41]. It can also provide a better option to differentiate between paragangliomas and other tumors, such as esthesioneuroblastoma, schwannoma, osteosarcoma, and plasmacytoma, due to their significantly lower uptake, but with limitations when it comes to meningioma and ectopic thyroid tissue, that both present a similar expression of SSTR [18]. Due to the excellent results it has shown in comparison with other functional imaging methods in identifying paragangliomas and their metastases, the European Association for Nuclear Medicine’s latest guidelines encourage the prioritization of ^68^Ga-DOTATE PET/CT examination, in place of ^18^F-FDA PET/CT or ^18^F-DOPA PET [42].

## 7. Treatment Options

In the past surgery was considered to be the gold standard and the only treatment option available for head and neck paragangliomas, but recent opinions have shifted toward a more individualized approach, due to postoperative morbidities and the development of radiotherapy and chemotherapy that showed impressive results on this type of tumors [1,3,19].

Some studies pointed out that the rate of recurrence varied with the therapeutical approach and the localization of the paraganglioma, pointing out that glomus jugular tumors had the highest chances of relapsing if treated by surgery, and rarely when radiotherapy was used [43]. Same study placed the carotid body paragangliomas on the opposite side, having the highest rate of recurrence if treated by radiotherapy, while surgery proved to give the best results [43].

### 7.1. Carotid Body Paraganglioma

Carotid body paragangliomas are the most common, representing almost 60% of all head and neck paragangliomas, typically originating from the adventitia of the carotid artery, in close proximity to the bifurcation, in the 5th and 6th life decade, being slow-growing masses [44]. Their clinical characteristics are represented by large pulsatile painless neck mass in 86% of the cases, which can be associated with tinnitus, ear pain, dysphonia, dysphagia, syncope, dizziness, and cranial nerve palsy [45]. The etiology of these types of paragangliomas is believed to be related to patients who suffer from chronic hypoxia, usually encountered in patients who live in high-altitude environments [45,46]. Malignant transformation of the tumors and the risk of metastases is around 6% [3].

When considering their evolution pattern, it has been proven that carotid body paragangliomas are indolent tumors, having an average growth of only 10.4% a year, encouraging a “watch and wait” approach for patients that are over 60 years old, asymptomatic, with small, benign tumors [47,48]. Waiting may also increase the risk of skull base and carotid artery invasion and predispose them to postoperative morbidities [2].

Any surgical decision regarding carotid body paragangliomas is centered on the Shamblin classification, which is used to assess the tumors and spread them in three major groups, as follows: Shamblin I tumors are small and easily resected; Shamblin II tumors are adherent or partially encase the carotid arteries, and Shamblin III tumors are large and intimately surround the carotid vessels, Table 1 [49]. Shamblin classification is used to predict the difficulty of the surgery. Shamblin I tumors can be easily dissected from the vessels, but in Shamblin II, the focal adventitia is partially infiltrated, therefore, dissection is difficult but the artery can be preserved, although the wall is damaged. Finally, in Shamblin III, it is impossible to reach a plane of dissection, so the artery might need to be sacrificed [50]. These have also been determined to assess the risk of stroke in correlation with Shamblin classification, and it has been revealed that Shamblin III tumors are associated with 60% risk, Shamblin II only 25% risk, and Shamblin I with 15% risk [45]. The Same study correlates the cranial nerve injuries with the aforementioned classification, concluding that 54% of them occurred in Shamblin III, 39% in Shamblin II, and only 7% in Shamblin I patients [45]. The most encountered nerve injuries are the temporary paresis of the hypoglossal and facial nerve, especially the mandibular branch, but with resolution within one month [51].

Blood loss has also been discussed when talking about carotid body paraganglioma surgery [51]. Chemoembolization was tried with good results, obtaining a total devascularization of the tumor, using either ethylene vinyl alcohol copolymer (EVOH) injected percutaneously into the tumor or by transarterial onyx embolization [52,53,54]. Precautions should still be taken, as it might increase the risk stroke, as a result of the preoperative embolization [55].

Although it has been guiding surgical decisions for many years, the Shamblin classification has its limitations, as it uses the anatomical and radiological criteria and leaves out the clinical one [56]. Carotid body paragangliomas that extend to the infratemporal fossa and involve the carotid canal or the jugular foramen are left out of the Shamblin classification [56]. Other classifications have been proposed, but studies showed that there is still no consensus on whether they bring a significant improvement or not [57].

When considering the decision to proceed to surgery, some studies showed that the presence of SDHB mutation should be taken into consideration, as it has been proven to have a poor disease-free survival than those with SDHD, although the size of the resected tumor was smaller [58]. The study showed that the resection of a tumor with positive SDHB mutation needs to be carefully planned, as it involves a higher risk of local relapses and metastases, and it proposes a more aggressive surgical treatment in association with preoperative embolization [58].

The gold standard for a successful operation is the complete resection of the tumor without permanent complications; radiotherapy, and chemotherapy have been reserved for Shamblin III tumors larger than 5 cm [2,30].

### 7.2. Temporal Bone Paraganglioma

Temporal bone paragangliomas (TBG) are the second most common, representing 20–30% of all head and neck paragangliomas [8]. Those originating from the Jacobson’s nerve, the tympanic branch of the glossopharyngeal nerve, and Arnold’s nerve, the auricular branch of the vagus nerve, are considered to be tympanomastoid paragangliomas, and the ones that have their origin from the paraganglia of the jugular bulb’s adventitia are called tympanojugular pargangliomas [8]. Temporal bone paraganglioma presents a tendency to invade the skull base, the tympanojugular is located around the tympanic canaliculus, while the tympanomastoid is usually found within the jugular foramen [13]. The incidence of the temporal bone paraganglioma was reported to be around 0.7 cases per 100,000 every year [8].

Clinical characteristics of a temporal bone paraganglioma are typically hearing loss and pulsatile tinnitus. In the case of a larger tumor, it might be associated with dysfunction of cranial nerves, most commonly IX and X. Still, it can also affect XI and XII, causing symptoms like hoarseness, dysphagia, shoulder weakness, and dysarthria [8,59]. Studies have revealed that more than 60% of all patients with temporal bone paragangliomas exhibit at least one cranial nerve palsy [31]. A complete head and neck examination is mandatory, as it can be diagnosticated by otoscopy, displaying a red mass localized posterior to the tympanic membrane [60]. The most utilized classifications of temporal bone Fisch classified temporal bone paragangliomas into four classes based on the extension of the tumor and location, A, B, C, and D as it follows: **Class A** (Glomus Tympanicum)—Limited to mesotympanum; **Class B** (Glomus hypotympanicum)—Limited to hypotympanum, mesotympanum, and mastoid without erosion of jugular bulb; **Class C**—Involvement and destruction of infra labyrinthine and apical compartments with subclasses **C1**—No invasion of carotid; destruction of jugular bulb/foramen; **C2**—Invasion of vertical carotid canal between foramen and bend; **C3**—Invasion along the horizontal carotid canal; **C4**—Invasion of foramen lacerum and along carotid into cavernous sinus [61]. **Class D** represents the intracranial extension (De, extradural; Di, intradural) with subclasses as it follows: **De1**—up to 2 cm discplacement and **De2** with more than 2 cm displacement; **Di1**—up to 2cm intradural displacement and **Di2**—more than 2 cm intradural displacement [61]. 

Glasscock-Jackson highlights two major types, Glomus Tympanicum and Glomus jugular, with four different subgroups each, without making the difference between the timpanojugular paraganglioma and timpanomastoid paraganglioma [62]. The glomus tympanicum is classified in 4 class as it follows: **I**—Small mass limited to the promontory; **II**—Tumor filling middle ear space; **III**—Tumor filling middle ear and extending into the mastoid and **IV**—Tumor filling middle ear, extending into the mastoid or through tympanic membrane to fill the external auditory canal; may extend anterior to the carotid [62]. The Glomus jugular also has 4 subclasses: **I**—Small tumor involving jugular bulb, middle ear, and mastoid; **II**—Tumor extending under internal auditory canal; may have intracranial canal extension (ICE); **III**—Tumor extending into petrous apex; may have ICE and **IV**—Tumor extending beyond petrous apex into clivus or infratemporal fossa; may have ICE [62].

Temporal bone paragangliomas are hypervascularized tumors; therefore, bleeding control is a key point when deciding the surgical treatment [8]. Methods for preoperative embolization of the tumor have been discussed, using different agents, such as cyanoacrylate glue (NBCA), polyvinyl alcohol (PVA), and ethylene vinyl alcohol (onyx) [63]. According to recent studies, polyvinyl alcohol seems to be the best option for embolization, in comparison with onyx, which proved to give multiple complications, most frequent being cranial neuropathies, due to common vascularization between jugular paraganglioma and facial nerve [64,65]. It should always be kept in mind that tumors that develop near the jugular foramen tend to create a plug-like phenomenon that might lead to venous hypertension; therefore, the embolization of the inferior petrosal sinus seemed to be of great aid when operating these types of tumors [63]. The main blood supply of the temporal bone paraganglioma is the posterior auricular artery, ascending pharyngeal artery, and occipital artery, and they should be embolized superselective within 24 h and 48 h prior to the operation for the best results [66]. It is recommended to limit the embolization for Fisch C and D tumors, which are the classes with the highest risk of bleeding and postoperative complications due to their advanced vascular invasion [8,63,67].

For many years, the only treatment for temporal bone paragangliomas was surgery, but with the latest advances in radiotherapy and radiology, the attitude has shifted to a more conservative management [8]. The therapeutical options we have at our disposal are surgery, radiotherapy, a watch-and-wait attitude, or any combination of them while taking into consideration different factors such as age, comorbidities, cranial nerves functionality, type of tumor and its extent, and the degree of involvement of the great vessels of the head and neck, such as the jugular vein and the carotid artery [68].

The latest studies have concluded that when it comes to temporal bone paragangliomas in the A and B Fisch classes, gross surgical resection is the treatment that offers the best result, with significant postoperative complications appearing in less than 1% of the patients [69,70]. The most encountered postoperative complications involve cranial nerve injuries, such as facial nerve palsy, lesion of the accessory nerve that lead to loss of sternocleidomastoid muscle and trapezius muscle functions, or the alteration of the mobility of the tongue by hypoglossal nerve injury [71]. Subtotal surgical resection in association with radiotherapy has proven to be the best choice for Fisch class C3, C4, and D, or Glasscock-Jackson stage III and IV, with a tumor control of 98% and no cranial nerve deficits [72].

The resection of tympanic paraganglioma has the goal of conserving hearing, and it should be carried out by a retroauricular approach with postoperative ossicular chain reconstruction [73].

In the case of the jugular paragangliomas, the conception has shifted towards achieving the best functional results; therefore, the Fisch technique involving an approach through the infratemporal fossa has been replaced with subtotal resection, using the intratympanic facial recess; in this way, preserving the auditory external canal and exposing of the internal carotid artery [73]. To assess if the patient is a candidate for surgery, three criteria have been suggested: the tumor must be limited to the infralabyrinthine chamber (Fisch B or C), the dissection of the tumor from the cochlea and inner ear must be possible, and the tumor must not extend further than the infratemporal fossa [73]. Other criteria to indicate the necessity of surgery have also been added, such as a young age, tumors that secrete catecholamine, facial paralysis, symptoms that suggest intracranial pressure from the tumor, the tumor keeps growing after radiation, malignant transformation, and the presence of lower cranial nerve functional deficits at presentation [8].

For Fisch C1 and C2, with or without Di or De extensions, complete removal of the tumor is possible, preventing functional deficits for lower cranial nerves, with less than 10% of new postoperative nerve palsies in cases with Di/De, and none in those without extensions [74].

For Fisch C3 and C4, an infratemporal fossa approach type A can be performed or in combination with infratemporal fossa approach type B to remove the tumor, while a trans-cochlear approach is useful if the tumor invades the foramen magnum, the clivus. or the occipital condyle [75]. Some studies on tympanojugular paragangliomas with Fisch C and D tumors have revealed postoperative complications to involve cranial nerve deficits that occur in 18%, with a percentage of 6.6% presenting facial nerve dysfunctionality, 2.2% encountering a worsening during the follow-up, and 5.3% with cerebrospinal fluid leakage [76].

A study of 53 patients with jugular paragangliomas with tumor staging C1 (7.5%), C2 (66%), C3 (22.6%), or C4 (3.7), showed 62.2% of the patients having an intracranial involvement, out of which 72.7% were intradural and 27.2% extradural [77]. By performing an infratemporal fossa aporach type-A (ITFA-A), a gross total removal was achieved in 90.7% of the cases, with recurrence found in five patients, one with C4Di2, one with C3Di2, and three with extradural invasion (C2, C2, C2De2) [77]. In 9.1% of cases belonging to C4Di1, C3Di2, C2Di2, and C1, a subtotal removal of the tumor was performed [77]. For C3Di2, a first stage ITFA-A was performed followed by the transcochlear D approach; 4 months later, tumoral residual was revealed at the follow-up [77]. In the case of a C4Di1 tumor. a partial removal of the tumor was performed followed by stereotacticsurgery [77]; 80.8% of the patients with no intracranial extension preserved their cranial nerve functions 1 year after surgery, in comparison with 70.1% with incranial extensions, which proves that surgery still provided good outcomes even in the most advanced cases [77]. In cases of Di3 tumors, palliative radiotherapy is prefered [8].

The latest studies advocate that for Fisch C and D classes, radiotherapy, including stereotactic radiosurgery, could be an important therapeutical choice, in some cases obtaining 98% tumor control with only 3% cranial nerve complication, in comparison with surgery that achieved 95% tumor control, but with a high price, with 67–100% rate of cranial nerve injury [8,69]. Radiotherapy represents an option for patients who refuse surgery, have many comorbidities that prevent the operation, elderly patients with no cranial nerve deficits at presentation after a subtotal resection of a large tumor, and for cases that involve the carotid artery, in which stenting is impossible and a subtotal removal was performed [8]. It must be highlighted that radiotherapy does not represent a curative treatment option, rather, it obtains a tumor control or a reduction of 10% to 25% in size [8]. Radiotherapy must not be recommended for young patients, large, secretory, and symptomatic tumors, or tumors with intracranial extension, as it may cause cerebral edema and intracranial hypertension, surgery being the sole option in these cases [8].

The watch-and-wait strategy is usually reserved for patients over 50 years old with a unilateral tumor, multiple comorbidities, a high risk of postoperative neuropathy with cranial nerve functions preserved or compensated, no vascular invasion, and the absence of SDHx-related genetic mutation [8,78].

### 7.3. Vagal Nerve Paraganglioma

Vagal nerve paragangliomas are the third in order of frequency, being accountable for almost 5% of all paragangliomas, with an incidence of 1 case per 100,000 per year [79]. A female predominance has been observed with a female-to-male ratio ranging from around 2.7:1 to 6:1 [80,81]. In most cases, they are benign tumors, but studies showed that they have a percentage between 6% and 19% to express malignant behavior, having the highest rate of malignancy among all head and neck paragangliomas [82]. Typical clinical presentation is represented by a lateral neck mass associated with hoarseness, and in some cases, pulsatile tinnitus, Horner syndrome, and shoulder weakness due to the encroachment of the cranial nerves IX, X, XI with its spinal accessory branch, and XII [31]. Due to the possible involvement of the recurrent branch, laryngoscopy is mandatory in assessing a patient with vagal nerve paraganglioma in order to highlight any vocal fold paralysis [60].

Surgery has been considered the only treatment method, its main goal being the complete removal of the tumor while preserving as much as possible the carotid artery, the jugular vein, and the cranial nerves, considering the fact that in only between 5% and 8% of the cases, the vagal nerve can be sparred, without any associated morbidities [83,84].

Being an incredible challenge to dissect the tumor without injuring the cranial nerves or the great vessels of the neck, the latest studies encourage a watch-and-wait strategy for young patients with no neurological deficit unless the tumor grows and starts associating nerve dysfunctions [79].

Surgery usually starts with a transcervical periauricular approach to the infratemporal fossa, with an extension superiorly via a transtemporal approach [60]. In most cases, complete removal of the vagal nerve is necessary, which results in permanent vocal fold paralysis, an ipsilateral pharynx numbness, and a unilateral velopharyngeal dysfunction due to a resulting pharyngeal plexus deficit [60].

In the case of a malignant vagal nerve paraganglioma, the therapeutical strategy is still a matter of debate, as it occurs extremely rarely, but most commonly, a surgical resection associated with postoperative radiotherapy is recommended [82].

Radiotherapy is the primary treatment for elderly patients who associate comorbidities that do not qualify them for surgery, after a watch-and-wait surveillance, with a growing tumor [80].

## 8. Malignancy

Paragangliomas of the head and neck are in their vast majority benign tumors, with only 6% to 19% developing metastases and showing a malignant behavior [1]. There are no specific criteria to establish whether a tumor is benign or malignant; therefore, the latest consensus of the World Health Organization considers all paragangliomas to be malignant [5]. Some characteristics might have the potential to indicate the possibility of a malignant tumor, such as a Ki67 higher than 3%, the presence of necrosis in the histopathological specimen, a tumor larger than 5 cm, and the identification of the SDHB mutation, but they are far for providing any certainty [5]. The most common places for metastases are the bones, cervical lymph nodes, lungs, liver, and thyroid [85]. The 5-year survival rate among patients with metastases varies greatly between 12% and 81%, and because of their rare rate, there is no standardized treatment [85]. A metastatic paraganglioma is characterized by the presence of metastases in nonchromaffin organs [86,87].

Due to their slow growth rate, there is no period of time for a specific follow-up, metastasis being discovered decades after the primary tumor was treated, so the patients should be under biochemical and radiological surveillance during their entire life [86].

Treatment options for metastatic paragangliomas include surgery, radiotherapy, chemotherapy, immunotherapy, and peptide therapy [10,86]. Studies indicate that to have a better chance to anticipate the malignant behavior or for curative purposes, lymph node dissection in groups IIa, IIb, and III in all patients with head and neck paragangliomas is advisable [86,88].

For cases in which distant metastases are discovered, with advanced disease or failure after iodine-131-MIBG therapy, chemotherapy is the treatment of choice, with agents like cyclophosphamide, vincristine, dacarbazine, sunitinib monotherapy, temozolomidecapecitabine or temozolomide, and thalidomide [86,89]. Other studies that take into consideration therapies for the somatostatin receptor–ligand, and tyrosine kinase inhibitors are under development, and results are yet to be seen [1].

A study consisting of 46 patients with metastatic paraganglioma, treated with peptide receptor radionuclide therapy (PRRT), out of which 12 had 90T-DOTATOC and 34 had 177Lu-DOTATATE, 5 cycles each, showed an average disease control rate (DCR) of 80.4%, with a partial response of 8.7%; 71.7% showing a stable disease and 19.6% with progressive disease [90]. Differences between the two agents seemed to be minimal, with a DCR of 75% for 90T-DOTATOC and 82.4% for 177Lu-DOTATATE [90]. The 9.2 GBq dosage of 90T-DOTATOC had a better DCR after 76 months, of 88.9%, while the 6.4 GBq only 33.3% [90]. 177Lu-DOTATATE, with a 15.9 GBq dosage, presented a DCR of 55% in comparison with 25.9 GBq, which obtained a 92% DCR [90]. One patient presented G1 toxicity and one with G2, the latter needing hemodialysis after more than 10 years from the starting point of the treatment [90].

Cabozantinib, an antiangiogenic multi-tyrosine kinase inhibitor, was tested on 17 patients with metastatic paraganglioma in a single-arm phase 2 trial, with a follow-up period of 25 months [91]. The dosage was 60 mg/day, with a response rate of 25.0% and seven grade 3 adverse effects encountered in 6 patients, proving that it might represent a solution for treating metastatic paragangliomas in the near future [91].

Surgery remains the primary treatment option for local progression of the tumor, associated with radiotherapy, with complete removal of the tumor, or just radiotherapy to slow down the growth in cases in which the resection is not possible [89].

## 9. Management of Multicentric Paragangliomas

Patients who exhibit bilateral paragangliomas should benefit from an individualized approach, as there is no specific algorithm, and multiple factors must be taken into consideration, such as age, comorbidities, size and location of the tumor, and neurologic function [60].

Bilateral vagal paraganglioma or jugular paraganglioma pose the biggest challenges as they involve a high risk of complications and failure; therefore, most recommendations are against any surgical treatment, replacing it with a watch-and-wait conduct or radiotherapy in case of neurological dysfunctions [60,92]. Another approach might be to operate one of the tumors, should it become symptomatic, starting with the largest one, and watch and scan the others, to prevent complications such as bilateral vagal nerve palsy [1].

A 3D-reconstruction might come in handy for patients with multiple paragangliomas, as it offers a better understanding of the relationships between the tumors and the nerves and vessels, reducing the chances of postoperative complications [93].

## 10. Prognosis

Paragangliomas of the head and neck are mostly benign tumors, having an overall survival rate after 5 years of 91% for those with no distant metastases [20]. On the opposite are the patients with malignant tumors, having a 76.8% in case of lymph node metastases, and 11.8% for those with distant extensions [94].

Although patients with benign head and neck paraganglioma have a great chance of survival, some of them might have their quality of life altered permanently, experiencing hoarseness due to vocal fold palsy after vagal nerve surgery or fatigue in case of ventilatory dysfunctions [95]. Individuals who have been diagnosed with metastatic paragangliomas tend to suffer from anxiety and depression [1].

Prolonged surveillance is mandatory. The British Skull Base Society proposes an MRI scan every 6 months initially, with an annual re-examination [96].

In cases of patients with secretory paragangliomas, metanephrines levels should be dosed from plasma and urine 2 to 6 weeks after surgery, to be completely sure that total resection has been achieved, and annual for those with a high risk of developing metastases, accompanied by a full MRI scan from skull base to pelvis [97].

There is no general consensus on the duration of surveillance. The British Skull Base Society recommends radiological scanning early in the first 3 years followed by reduced intervals, while the European Society of Endocrinology advises a 10-year minimum period, with lifelong observation for patients with risk factors or with genetic predisposition [96,97].

## 11. Conclusions

Paragangliomas of the head and neck represent extremely rare and, therefore, challenging pathologies. They are benign in their vast majority, but some of them turned out to be malignant, and the lack of specific markers to make the difference, except the identification of metastases, poses, possibly, the biggest management challenge. The aim of this article is to summarize and more comprehensively present the latest data and guidelines that, in our opinion, every head and neck surgeon should know when managing this type of tumor.

There is still a lot to debate about head and neck paragangliomas. The fact that they have a high rate of genetic inheritance has been proven, but clear correlations between gene mutation and the behavior of paragangliomas remain to be established.

Another aspect that needs more attention in the future is the development of a general classification of paragangliomas, which should result from the harmonization of the existing ones so that it should offer a clearer guideline of treatment for paragangliomas. The current existing classifications, although proved to be effective, clearly have limitations.

Imaging has developed significantly in the past year, especially by introducing functional radiology, but the costs of these new techniques are still high, and in many cases, it prohibits access to them. Making them more affordable for patients with paragangliomas will bring a significant improvement in treatment and long-term follow-up.

The lack of consensus among the practitioners regarding the necessity of surgery and the surgical approach is a result of the rarity of these tumors and the lack of experience in treating them.

With the development of efficient conservative therapies, such as radiotherapy, proton therapy, and chemotherapy, studies should be carried on to harmonize all treatment methods and issue a standardized guideline.

## Figures and Tables

**Table 1:** Shamblin classification of carotid body paragangliomas [49].

Grade	Anatomical Description
Shamblin I	Tumors are localized and easily resected without vessel injury
Shamblin II	Tumors adherent or partially surrounding the carotid vessels but with dissection still possible
Shamblin III	Tumors intimately surround or encase the carotid vessels with impossible dissection.
